# Gastrointestinal stromal tumor of the anal canal: an unusual presentation

**DOI:** 10.1186/1477-7819-5-20

**Published:** 2007-02-16

**Authors:** Giuseppe R Nigri, Mario Dente, Stefano Valabrega, Paolo Aurello, Francesco D'Angelo, Giuseppe Montrone, Giorgio Ercolani, Giovanni Ramacciato

**Affiliations:** 1 Department of Surgery, University of Rome "La Sapienza", 2^nd ^School of Medicine, Sant'Andrea Hospital, Rome, Italy; 2 Department of Pathology, University of Rome "La Sapienza", 2^nd ^School of Medicine, Sant'Andrea Hospital, Rome, Italy

## Abstract

**Background:**

Gastrointestinal stromal tumors (GIST) of the stomach are the most frequent followed by those of the intestinal tract, while colon and rectum represent rare sites. GIST of the anal canal are extremely rare. They have been studied along with GIST of the rectum, as a single entity, and along with them they represent 5% of GIST. GIST arising from the anal canal account for only 2%–8% of the anorectal GIST. Thus anal GIST must be considered an exceptional case.

**Case presentation:**

A 78-year-old man was referred to our Institution for an anal mass, in absence of any symptom. The patient was treated by local excision. An histological diagnosis of a low grade GIST was made. No further treatment was necessary. No local recurrence of distant metastases were found at follow-up.

**Conclusion:**

At the moment, only ten cases of c-kit positive anal GIST are reported in the literature. These few data are not sufficient to establish a widely accepted approach for this neoplasia.

We recommend to perform an initial local excision, to define the risk of aggressive behavior and the resection margins and proceed to a more aggressive treatment, if the GIST belongs to high or very high risk group. The role of adjuvant therapy is still uncertain. Although inhibitors of tyrosine-kinase receptor needs further studies before their routine use, their role in case of distant or local recurrence has been accepted. Patients' close follow up is mandatory to disclose as soon as possible local recurrences or metastases.

## Background

Gastrointestinal stromal tumors (GIST) represent the most frequent mesenchymal neoplasm of the GI tract. As reported by Nilsson et al., epidemiological data virtually are non existent regarding the true incidence and prevalence of GIST[1]. This is due to the previous lack of well defined pathologic criteria for GIST, varying nomenclature for GIST over the past few decades, and the finding that nearly 60% of all GIST have been diagnosed as benign tumors or tumors of uncertain malignant potential, thus they are not reported to national cancer registries[1]. Therefore, Nilsson et al. analyzed the incidence and prevalence of GIST in a defined population, in a province of western Sweden. In that region the annual incidence of clinically detected GIST was estimated 14.5 per million inhabitants and the prevalence was 22.2 per million for very low risk GIST, 51.9 per million for low risk, 24.2 per million for intermediate risk, 22.2 per million for high risk and 8.7 per million for malignant GIST[1].

GIST are defined as mesenchymal neoplasm expressing KIT protein, driven by *KIT *or *PDGFR*α (platelet derived growth factor alpha) mutations[2]. They are regarded as derived from interstitial cells of Cajal (ICC). ICC are pacemaker cells that regulates peristalsis and have immunophenotypic and ultrastructural features of both smooth muscle and neural differentiation in varying degrees. ICC are KIT positive cells. Activation of *KIT *by mutations, causes Cajal cell proliferation and GIST[3].

Most gastrointestinal stromal tumor (GIST) develop in the stomach (50–60%), followed by small intestine (30–40%), colon (7%) and esophagus (1%). Anal canal represents an extremely rare site of GIST[4]. Due to the rarity of both rectal and anal GIST, just a few data exist about their single incidence. They are often classified as anorectal stromal tumors representing the 5% of all GIST[4,5]. We present a case of anal GIST, treated by local excision, in order to discuss diagnosis, surgical treatment and adjuvant therapy of these rare lesions.

## Case presentation

**Figure 1:**
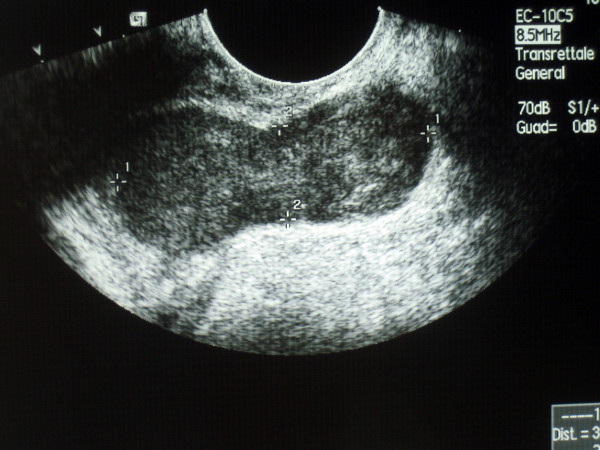
Endoanal ultrasonography (longitudinal plain) shows a bilobate, circumscribed 4 × 2 cm mass.

**Figure 2:**
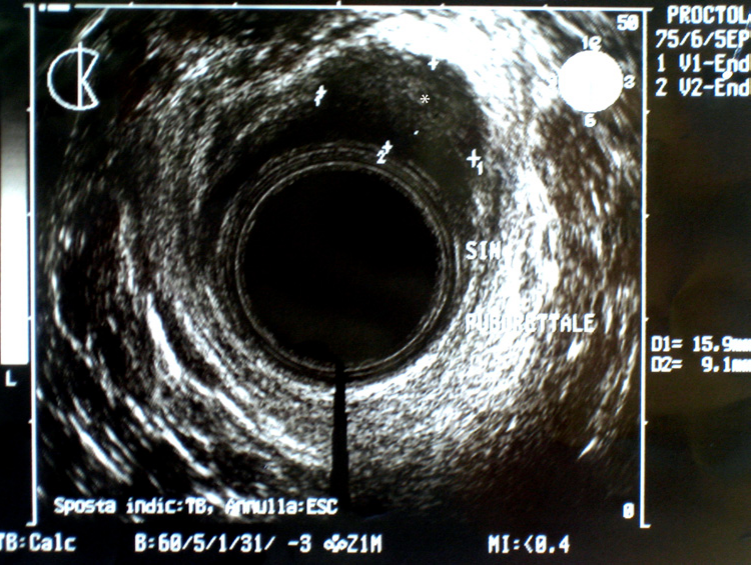
Endoanal ultrasonography (transverse plain) shows the mass (*) located along the left anterior aspect of the anal canal.

**Figure 3:**
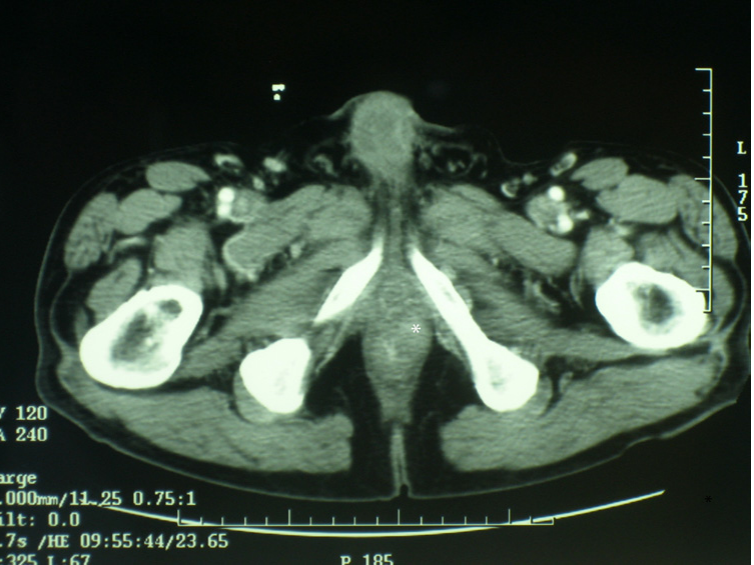
CT scan shows the mass (*) along the left anterior aspect of the anal canal.

A 78-year-old man was referred to our Institution for the presence of an anal mass accidentally discovered during a routine physical exam. Past medical history was significant for cancer of the right lung, treated with right pneumonectomy 1 year before. Routine blood test were within normal limits as well as common neoplastic markers. The rectal exam showed a well defined mass on the left-anterior aspect of the anal canal, beginning at 1 cm from anal verge and extending cranially for about 4 cm. Endoanal ultrasonography confirmed the presence of a 4 × 2 cm mass in the thickness of the sphincteric muscles (Fig 1 and 2). Total body CT scan confirmed the presence of the mass and did not show any lymph node enlargement in the proximity or distant metastases. The mass appeared circumscribed and not infiltrating the surrounding tissues (Fig. 3).

**Figure 4:**
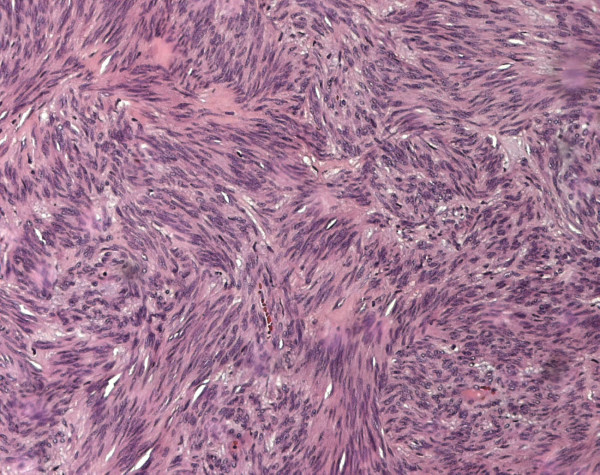
Fascicular arrangement of spindle cells with prominent nuclear palisading and perinuclear cytoplasmic vacuoles (×200).

**Figure 5:**
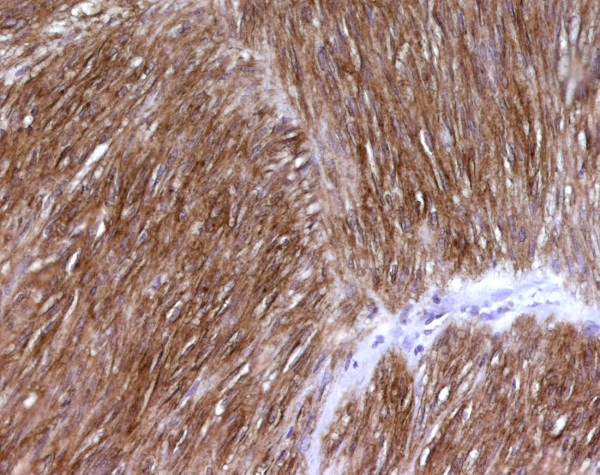
KIT staining in GIST. Cytoplasmic and perinuclear strongly positive tumour cells (×200).

The patient was brought to the operating room, placed in jack-knife position, and a local excision was carried out, resecting just a small amount of fibers of the anal sphincter. This has been possible since the mass was well capsulated and not firmly adherent to the surrounding structures. Gross pathological examination showed a 3.5 × 2 × 1.2 cm fibrous-elastic mass. Histological examination showed a proliferation of densely packed spindle cells, with prominent nuclear palisading (Fig. 4). Nuclear atypia was absent and mitotic count was of 4 mitosis/50HPF. Neoplastic cells showed diffuse and marked cytoplasmic positivity for KIT protein and CD34 in the majority of cells (Fig. 5). Neoplastic cells were negative for desmin that stained residual smooth muscle fibers of bowel wall at the margins of the neoplasm. A diagnosis of GIST, with low risk aggressive behavior was made (Table 1)[6], therefore no further treatment was necessary.

**Table 1:** Proposed approach for defining risk of aggressive behaviour in GIST [9]

	Size	Mitotic Count
Very low risk	< 2 cm	< 5/50 HPF
Low risk	2–5 cm	< 5/50 HPF
Intermediate risk	< 5 cm	6–10/50 HPF
	5–10 cm	< 5/50 HPF
High risk	> 5 cm	> 5/50 HPF
	> 10 cm	Any mitotic rate
	Any size	> 10/50 HPF

Postoperative course was uneventful. No implications on anal continence were observed and the patient was discharged on postoperative day 3. The patient underwent follow up at 6 and at 12 months, and rectal ultrasonography as well as CT scan did not show local recurrence or distant spread.

## Discussion

GIST are the most common mesenchymal neoplasms of the digestive tract. They are found more often in the stomach (60–70%) and less frequently in the small intestine (30%), while both the rectum and anus are extremely rare locations with an incidence of 5% of all gastrointestinal stromal tumors [4]; indeed anal GIST is a rarity representing only the 3% of all anorectal mesenchymal tumors [7].

Mutational status of c-KIT and PDGFRα genes are the basis for the diagnosis of this neoplasia, an it represents the criteria for surgical therapy, expected chemotherapy response and clinical outcomes. In fact, most of GIST express c-KIT [8,9]. The KIT protein (or CD117 antigen), a proto-oncogene, is a transmembrane tyrosine kinase receptor for a growth factor termed stem cell factor (SCF). Mutations of *KIT *gene causes constitutional activation of the kinase ligand-independent [3]. In a small percentage of cases a mutations in another tyrosine-kinase receptor (PDGFRα) has been demonstrated. Inhibitor of tyrosine-kinase receptor as imatinib mesylate (STI-571, Gleevec; Novartis, Switzerland), represents the target therapy for local or distant recurrence after surgical resection in GIST[10,11]. The effect of tyrosine-kinase inhibitor are affected by the exon mutations on *KIT *gene. Several studies reported clinical evidence of tumor response to imatinib, ranging from 12 % and 70% in cases of exon 9 and exon 11 mutations of the *C-KIT *gene respectively[12].

Although prediction of clinical outcome has been extensively studied [13-15] the widely accepted criteria to predict the malignancy of GIST are the mitotic activity (>5 mitotic figures per 50 × high power field) and the tumor size (>5 cm) (Table 1)[6]. But also in case of a very low risk lesion, Fletcher et al. stressed its spreading potential, admitting the presence of still unknown malignant factors[6]. Factors as mucosal invasion and tumor necrosis have found to be related to increased risk of aggressive behavior, but their clinic value remains uncertain. It should be noted that the guidelines proposed by Fletcher et al. recommend categorizing GIST into risk categories, emphasizing that no lesion can be definitely labeled as benign[6,16].

In addition the GIST susceptibility to metastasize via bloodstream, and to relapse as local recurrence, makes the surgical treatment controversial regarding the extent of resection. Authors who treated rectal GIST by an abdominoperineal resection reported a low local recurrence, with no improvement in incidence of distant metastases and overall survival rate[13].

We recommend, in case of anal GIST, to perform the less extensive excision that achieves the essential R0 resection and defines the aggressive risk grade, followed by a Miles abdominoperineal resection when histopathological diagnosis displays a tumor size >5 cm together with

Tan et al reported a total of 16 cases of anal GIST in the literature from 1966 to 2001[17]. However, the Authors enrolled in their review all cases in literature, without distinguishing GIST from the other mesenchymal stromal tumors and describing symptoms, treatment and outcomes of anorectal stromal tumors all together[5,7,18-22]. We reviewed the literature focusing only on c-kit positive anal GIST. We found three published papers, describing a total of nine c-kit positive anal GIST. Miettinen et Tworek did not focus on anal GIST features, dealing with both rectal and anal GIST as an homogeneous group, while Vidarsdottir reports an anal GIST, previously diagnosed as anal sarcoma in 1987[5,7,23]. Therefore, at this moment, only ten cases of c-kit positive anal GIST are reported in the literature.

## Conclusion

At the moment, only ten cases of c-kit positive anal GIST are reported in the literature. These few data are not sufficient to establish a widely accepted approach for this neoplasia.

We recommend to perform an initial local excision, to define the risk of aggressive behavior and the involvement of the resection margins. The margin positivity (R1) should indicate the need of a more aggressive treatment, such as, in selected cases, abdomino-perineal resection, especially if the tumor belongs to high or very high risk group. The role of adjuvant therapy is still uncertain. Although inhibitors of tyrosine-kinase receptor needs further studies before using them routinely as adjuvant therapy, their role in case of distant or local recurrence has been accepted. Patients' close follow up is mandatory to disclose as soon as possible local recurrences or metastases.

## Conflict of interest statement

The author(s) declare that they have no competing interests.

## Authors' contributions

**GN **designed the study, drafted and revised the manuscript

**MD **carried out the data and picture acquisition and participated in the writing process

**SV, PA, FD **performed bibliographic research and participated in manuscript revision process.

**GM **performed histologic assessment of the lesion.

**GR **participated in the editing process.

All authors read and approved the final manuscript.
